# Microbiome dynamics associated with the infection of grey field slugs by the biocontrol nematode *Phasmarhabditis hermaphrodita*

**DOI:** 10.3389/fmicb.2025.1619231

**Published:** 2025-08-20

**Authors:** Anh D. Ha, Dana K. Howe, Andrew J. Colton, Rory J. Mc Donnell, Dee R. Denver

**Affiliations:** ^1^Department of Integrative Biology, Oregon State University, Corvallis, OR, United States; ^2^Department of Crop and Soil Science, Oregon State University, Corvallis, OR, United States

**Keywords:** microbiome, *Pseudomonas*, *Phasmarhabditis hermaphrodita*, nematode, slug, infection assay, biocontrol.

## Abstract

The facultative-parasitic nematode *Phasmarhabditis hermaphrodita* has been used for many years as a biological control agent targeting slug pests. During the nematode’s infection process, the presence of certain bacteria has been suggested to affect the pathogenicity and efficiency of the nematodes in killing slugs, though the potential roles of different bacteria in affecting host-infection by nematodes remain unclear. In this study, we examined three experimental *P. hermaphrodita* populations cultured with three different bacteria: 1) *Escherichia coli* (EC), 2) a newly isolated *Pseudomonas* sp. strain (PS) that co-cultured with a *P. hermaphrodita* strain found in Oregon, USA, and 3) the original complex bacterial community (BC) associated with the nematode. For each treatment, we evaluated the pathogenicity of *P. hermaphrodita* at low and high concentrations towards adult grey field slug *Deroceras reticulatum* and investigated changes in the nematode microbiome structure before and after slug infection. Slugs exposed to EC, of both low and high concentration treatments, survived significantly longer than slugs exposed to PS high and BC high. Slugs in the BC low treatment survived significantly longer compared to BC high, but significantly shorter compared to EC high. We identified a wide variety of taxa components (82 genera) in the community associated with the nematode pre-infection, most of which were of low abundance. In all bacterial treatments post-infection, the number of genera almost quadrupled and the abundance of these taxa changed remarkably, although the taxa with the highest abundance remained stable. We also observed four *Pseudomonas* amplicon sequence variants (ASVs) that increased in abundance after slug infection in the *Pseudomonas* treatment. This finding suggests these taxa may play a role in the infection process, potentially acting as opportunistic pathogens, or facilitating infection progression through providing nematodes with a favorable food source, or contributing directly to the nematode’s virulence.

## Introduction

Invasive pest gastropod species such as the grey field slug, *Deroceras reticulatum* (Mollusca: Gastropoda), are among the most widespread and damaging pests of agricultural and horticultural production systems, causing quality reductions and yield loss on a broad range of crops including wheat, corn, legumes, vegetables, and fruits (Godan, 1983; Barker, 2002; Mc Donnell et al., 2024; Anderson et al., 2011). Slug pests have also been documented to vector several plant and human pathogens, such as *Alternaria brassicicola* (causing black spot disease in brassicas), *Escherichia coli* (causing food poisoning) (Mc Donnell et al., 2024), and *Angiostrongylus cantonensis*, the causal agent of the potentially lethal eosinophilic meningitis (Centers for Disease Control and Prevention, 2023). Conventional slug control methods primarily rely on chemical solutions, using active molluscicide ingredients such as metaldehyde, iron phosphate, and carbamates (Barker, 2002; Mc Donnell and Anderson, 2017). However, environmental concerns exist regarding the potential for these chemical molluscicides to harm non-target organisms (Aktar et al., 2009; UK Department for Environment, 2020; Tudi et al., 2021). Furthermore, there is evidence that some pest snail species may develop resistance to certain molluscicide compounds (Adomaitis et al., 2022). Therefore, alternative slug control strategies, such as biological control approaches, are increasingly common in integrated pest management systems.

*Phasmarhabditis hermaphrodita* is a facultatively parasitic, rhabditid nematode species that infects a variety of slug and snail species. The potential of *P. hermaphrodita* for biocontrol against slugs was realized as early as 1988, when it was confirmed to be highly virulent against a wide range of pest gastropods (Wilson et al., 1993; Rae et al., 2007). The nematode was sold commercially under the trade name Nemaslug^®^ in Europe for over 30 years; still, it is not currently available for commercial purchase in the United States, due to regulatory concerns regarding incomplete information about the effects of this nematode on native North America gastropod species. Recent work by our research team demonstrated that *P. hermaphrodita* infects and kills *Monadenia fidelis*, a non-target snail species endemic to the Pacific Northwest of North America (Denver et al., 2024). Discoveries of *P. hermaphrodita* in California (De Ley et al., 2014) and subsequently in Oregon (Mc Donnell et al., 2018; Howe et al., 2020) motivated further exploration of this nematode as a potential biocontrol agent for slugs in the United States.

Regarding the mechanism of infection, *P. hermaphrodita* shares some similarities with the well-studied entomopathogenic nematodes (EPNs) of the genera *Steinernema* and *Heterorhabditis*, which have been commonly used as biological control agents of insect herbivores (Lacey and Georgis, 2012). The infective juvenile-stage of *P. hermaphrodita* lives freely in the soil and gains entrance to hosts upon exposure, usually through the mantle cavity. Once inside, infective juveniles develop into self-fertilizing hermaphroditic adults, reproduce, and the infection spreads to the entire body of the slug host, eventually causing its death within 4–21 days. The nematodes continue to consume the slug carcass until the food source is depleted, and their new infective juveniles again move into soil to search for new susceptible hosts (Tan and Grewal, 2001a; Rae et al., 2007).

Despite sharing many similarities in the life cycle with the EPNs of the genera *Steinernema* and *Heterorhabditis*, which, respectively, engage in obligate, specific mutualisms with *Xenorhabdus* and *Photorhabdus* bacteria (Forst et al., 1997; Sicard et al., 2004), *P. hermaphrodita* has a more uncertain and understudied relationship with its bacterial associates. The scientific literature focusing on the role of bacteria in the lifestyle, virulence, and efficacy of *P. hermaphrodita* presents inconsistent findings. In searching for a bacterium suitable for industrial-scale production of *P. hermaphrodita* as a commercial bio-molluscicide, Wilson and colleagues selected *Moraxella osloensis* (Wilson et al., 1995a, 1995b), which was later employed in the widely used Nemaslug^®^ product. However, they also observed that *P. hermaphrodita* could feed on and carry out slug infection with many other bacterial partners, including *Pseudomonas fluorescens* and *Pseudomonas paucimobilis*. Direct injection of *M. osloensis* into slugs did not cause significant mortality in the mollusk hosts (Wilson et al., 1995a, 1995b). On the contrary, Tan and Grewal proposed that the sole agent responsible for pathogenicity was *M. osloensis* by producing an endotoxin, and *P. hermaphrodita* served merely as a vector that transported the virulent bacterium into the shell cavity of the slug (Tan and Grewal, 2001b, 2002). Meanwhile, other studies suggested that *P. hermaphrodita* lacked a specific obligate partner in killing the slugs, and bacteria did not have a discernible influence on virulence (Rae et al., 2010; Sheehy et al., 2022). The precise roles of nematodes and bacteria in slug infection remain unclear, hindering the optimization of biocontrol methods using this nematode.

In this study, we aimed to investigate the intricate relationships between the nematode *P. hermaphrodita* and bacterial partners during slug infections, specifically whether different bacterial partners influence the virulence and pathogenicity of the nematode. We hypothesized that (1) *P. hermaphrodita*’s virulence would vary depending on the specific bacteria it was reared with, and (2) the pre-infection bacterial associate would differentially influence the nematode’s post-infection microbiome composition. To test these hypotheses, we conducted a set of slug infection trials to evaluate the pathogenicity of *P. hermaphrodita* nematodes reared under three distinct bacterial conditions:

1) *Escherichia coli* strain OP50. This non-pathogenic bacterial strain is commonly used as a standard laboratory food source for nematodes such as *Caenorhabditis elegans*, and has proven viable for rearing the nematode *P. hermaphrodita* (Andrus and Rae, 2019). Importantly, *E. coli*, including pathogenic strains, has demonstrated no virulence or lethality towards grey field slugs or other terrestrial gastropods (Sproston et al., 2006). Therefore, its inclusion would allow for a baseline assessment of nematode pathogenicity with a neutral bacterial partner, distinct from bacteria naturally associated with *P. hermaphrodita*.
2) One *Pseudomonas* sp. strain, isolated from the microbial community naturally associated with a *P. hermaphrodita* nematode collected in Oregon. *Pseudomonas* bacteria have also been found to be a viable food source for *P. hermaphrodita* (Andrus and Rae, 2019; Rae et al., 2023), and have exhibited virulence to slugs on its own (Wilson et al., 1994). Using this specific isolate allowed us to examine the impact of a naturally associated, potentially co-evolved bacterial partner on nematode virulence and preliminarily assess its potential as a bacterial partner for slug biocontrol.
3) The complex bacterial community naturally co-cultured with *P. hermaphrodita* found in Oregon. Utilizing the nematode’s original microbial associates allows us to assess its pathogenicity in a context that more closely mimics its natural ecological setting, and to understand how this diverse microbial community collectively influences infectivity, compared to single bacterial species effects.

Our results contribute insights into the dynamics of microbiomes during nematode-slug infection processes. We demonstrate that nematode’s bacterial consortia undergo distinct changes following infection, and the specific changes in bacterial community composition are influenced by the type of co-infected bacteria. This further supports the conclusion that *P. hermaphrodita* can successfully infect and kill slug hosts in association with various bacterial partners, and critically, highlights that specific bacterial associates may influence the infection outcomes, not merely through their presence, but by actively shaping the nematode’s microbial landscape.

## Materials and methods

### Culturing of nematodes and bacteria

The *P. hermaphrodita* strain used in this study (DL 309) was isolated from a dead slug collected in Oregon, USA (Howe et al., 2020). Nematodes were maintained in the lab on standard nutrient growth media (NGM) agar plates with co-cultured bacteria as a food source. NGM plates were prepared using autoclave-sterilized media, equipment, and sterile plastic plates (VWR International, Lutterworth, UK) and seeded with the appropriate bacterial cultures. Prior to our study, nematodes were bleached with sodium hypochlorite 0.25 M solution following standard worm bleaching protocol (Stiernagle, 2006) to minimize bacterial carry-over from previous culturing conditions. Nematodes were monitored on plain NGM agar plates for 24 h to ensure no bacterial growth, then harvested and washed once with sterile M9 buffer and twice with molecular biology grade water. Next, nematodes were transferred onto fresh NGM plates (Stiernagle, 2006) seeded with one of the three designated bacterial food sources: *E. coli* OP50 (EC)*, Pseudomonas* sp. (PS)., or the original bacterial community (BC) associated with the nematodes. The *E. coli* OP50 culture was obtained from the *Caenorhabditis* Genetics Center at the University of Minnesota. The *Pseudomonas* strain was isolated from the original bacterial community that co-cultured with a *P. hermaphrodita* sampled at the Oregon State University campus (Mc Donnell et al., 2018). We confirmed the genus identity of the strain with 16S rRNA and *rpoB* gene sequencing.

### Infection assay procedure

#### Experimental setup

Grey field slugs (*D. reticulatum*) were collected from several ryegrass seed production fields around Tangent, Linn County, Oregon, 1 day prior to the experiment and maintained in a growth chamber (Thermo Scientific Precision Model 818) at 18°C and with a 12-h photoperiod. The infection assay was performed in 16 oz. plastic round containers (13.8 cm in height x 11 cm in diameter), each containing 25 g of sterilized, damp EarthGro^®^ topsoil. Soil was dampened in each container by adding 10 mL of deionized water and mixing thoroughly. The containers were stored in the same growth chamber.

Our infectivity assay followed the approach described by Mc Donnell et al. (2020). Immediately before the trial, nematodes were harvested from NGM plates and washed once with sterile M9 buffer and twice with molecular biology grade water. The number of nematodes was estimated by counting 1 mL sub-samples, and the nematodes were then resuspended in 5 mL of sterile water and distributed into 10 replicates per nematode-bacteria combination: five with a concentration of ~8,000 nematodes/ml (‘high dose’) and five with a concentration of ~4,000 nematodes/ml (‘low dose’). Nematodes were pipetted onto the soil to the final concentration of ~210 worms/cm^2^ for low dose, or ~420 worms/cm^2^ for high dose. In total, 30 containers were set up [3 combinations x 2 doses x 5 replicates = 30]. Six healthy, mature adult *D. reticulatum* slugs (>100 mg) were added to each of the 30 containers. In our previous pilot studies, no signs of aggression were documented with this number of slugs cohabiting in the same container. Five additional containers, each holding six slugs and no nematodes were set up as negative controls. All slugs were fed with sterilized organic carrots during the course of the experiment.

The slugs were monitored daily for symptoms of nematode infection (e.g., swelling of the mantle, emaciation of the slug body, exposure of the internal shell, and nematodes visible on the slug body) and mortality for 15 days. Lethal Time 50 (LT50) and fiducial limits (95%) were calculated for the slug survival data using probit analysis. Abbott’s formula was used to correct for control mortality. Statistically significant differences (*p* < 0.05) exist when there was no overlap between the 95% fiducial limits. All analyses were carried out using IBM^®^ SPSS^®^ Version 24.

#### Collection of nematode-bacteria samples before and after infection

For each of the three bacteria examined in this study (EC, PS, BC), three biological replicates were collected, both before and after host slug infection. Prior to infection, we collected nematode samples in a fixed volume of 200 μL nematode suspension in sterile M9 solution. Immediately before the infection assay, three replicates of nematode suspensions at high concentration (8,000 nematodes/ml) were taken out from each of the three combinations, totaling nine samples. The nematodes were washed once with M9 buffer, and then twice with distilled water. We also included twelve negative controls to assess the contamination introduced during the preparation procedure and sequencing: three samples of the M9 buffer, three samples of the water used during the infection assay step, three samples of water used during the nematode washing step, and three DNA Extraction kit blanks. The nine pre-infection nematode samples (hereafter referred to as “PreInf” samples) and twelve negative controls were stored at −80°C immediately after collecting.

After host slug infection, nematodes and bacteria from the high-dose nematode treatment were collected in three biological replicates. For each of the three bacterial treatments, we collected three slug carcasses, each from a separate container, to extract their nematode populations. Immediately following the death of a slug, the carcass was submerged in sterile M9 solution to elute nematodes. Collected nematodes were washed once with M9 buffer and then twice with molecular biology grade water (VWR International, Lutterworth, UK). The nematode samples (hereafter referred to as “PostInf” samples) were then stored at −80°C until DNA extraction. The simplified naming scheme for the six different bacterial treatment types used hereinafter is summarized in Supplementary Table S1.

### DNA isolation, 16S rRNA amplification and sequencing

Prior to DNA isolation, nematode samples were homogenized thoroughly by bead beating. We extracted DNA from the homogenate and from the negative control samples using the PowerSoil DNA Extraction kit (MOBIO, Carlsbad, CA, USA). DNA extractions were then quantified using a fluorescent plate reader at the OSU Center for Genome Research and Biocomputing (CGRB).

16S library preparation, amplification, and sequencing were conducted by the OSU CGRB. Amplicon libraries were prepared following the standard protocol by Illumina (Illumina, Inc., 2013). Primers targeting the V3-V4 region (forward: 5’TCGTCGGCAGCGTCAGATGTG TATAAGAGACAGCCTACGGGNGGC WGCAG3’; reverse: 5’GTCTCGTGGGCTCGGAGATGTG TATAAGAGACAGGACTACHVGGGT ATCTAATCC 3′) were used for PCR amplification (Klindworth et al., 2013). Subsequently, the PCR products were processed through clean-up, index PCR, library quantification, normalization, and pooling. Libraries were sequenced using paired-end MiSeq v2 Nano 300 bp platform (Illumina, USA).

### DNA sequence data processing

We used the DADA2 pipeline (Callahan et al., 2016) to trim reads, merge paired reads, denoise, quality filter, and infer amplicon sequence variants (ASVs). ASVs represent exact 16S rRNA gene sequence variants resolved to the level of single-nucleotide dissimilarity. Compared to the commonly used operational taxonomic units (OTUs) with a threshold of nucleotide difference of 97%, ASVs have been shown to offer better reproducibility, reusability, and comprehensiveness in 16S microbiome analyses (Callahan et al., 2017).

Phylogenetic analyses and representations of the ASVs were constructed using MAFFT alignment (Katoh et al., 2002) and FastTree 2 (Price et al., 2010). To assign taxonomy to the ASVs, we utilized a self-trained classifier, trained on the Silva Project’s database (release 132) (Quast et al., 2013). Putative contaminant sequences were identified and filtered out of sample data using the R package decontam (Davis et al., 2018). ASVs were considered a contaminant if they were more prevalent in negative controls than in positive samples and/or their frequency significantly varied inversely with sample DNA concentration (probability threshold *p* < 0.1).

We utilized the Phylum, Genus, and ASV levels as units for subsequent taxa analyses. Since different taxonomic classifiers trained on different databases may differ at lower taxonomic ranks (Balvočiūtė and Huson, 2017), the Phylum level was chosen as the main unit for taxa analysis; however, the Genus and ASV level were also selected for a finer resolution and subsequent composition analysis.

### *α*-diversity analyses

We used four metrics: Chao1, Shannon Index, Inverse Simpson, and Faith’s Phylogenetic Diversity (PD) to compare and evaluate *α*-diversity from different approaches. Chao1 index is an estimator of species richness, i.e., the expected total number of OTUs/ASVs in the sample given all the species were identified. It weighs the low abundance taxa (i.e., only singletons and doubletons) to infer the number of missing species (Chao, 1984). The Shannon Index estimates the overall richness and also the evenness/uniformity between the taxa present in the sample (Spellerberg and Fedor, 2003). Also focusing on the species richness and evenness, however, the Inverse Simpson index emphasizes more on species evenness (Simpson, 1949), while the Shannon Index gives greater weight to the species richness. Faith’s PD metric considers the phylogeny of taxa to estimate diversity and is proportional to how much of the OTU/ASV phylogenetic tree is covered by the taxa present (Faith, 1992).

To calculate the Shannon Index and Inverse Simpson, the ASV table was rarefied ten times to a depth of 100,000 reads to lessen the random biases introduced by random subsampling. Rarefying the ASV table to a fixed number of reads per sample is necessary for calculations of the Shannon Index and Inverse Simpson, as these two abundance-based indices could be substantially affected by the differences in the total number of reads between samples. The two indices were calculated for each of the ten rarefied tables using the package phyloseq (McMurdie and Holmes, 2013), and the mean values were used for *α*-diversity analysis. As for Chao1, which depends on low-abundance taxa, the ASV table was not rarefied to retain all ‘rare’ taxa. The Faith’s PD index was calculated using the package picante (Kembel et al., 2010). We assess the significance of the differences among the bacteria-nematode combinations at the same time point using Kruskal-Wallis tests. Mann–Whitney U tests or paired t-tests were applied, as appropriate, to test for the difference between the two time points before and after infection. The normality of diversity values was assessed using the Shapiro–Wilk normality test.

### *β*-diversity analyses

To assess the difference in the microbial community between samples, we used four β-diversity metrics: two abundance-weighted β-diversity indices (Bray–Curtis dissimilarity and weighted UniFrac) and two indices based on the species (ASV) presence-absence matrix (Jaccard distance and unweighted UniFrac). While Bray-Curtis and Jaccard are non-phylogenetic metrics, weighted and unweighted UniFrac also take into account the phylogeny of the bacterial community composition to assess differences between samples. Specifically, UniFrac distances are based on the sum of branch length shared between samples on the phylogenetic tree constructed from all the 16S rRNA sequences from all communities of interest. To test whether the groups differ significantly from each other, we conducted the permutational multivariate analysis of variance (PERMANOVA) test using the package vegan (Oksanen et al., 2012).

## Results

### Pathogenicity and efficacy of *P. hermaphrodita* in killing slugs

**Figure 1:**
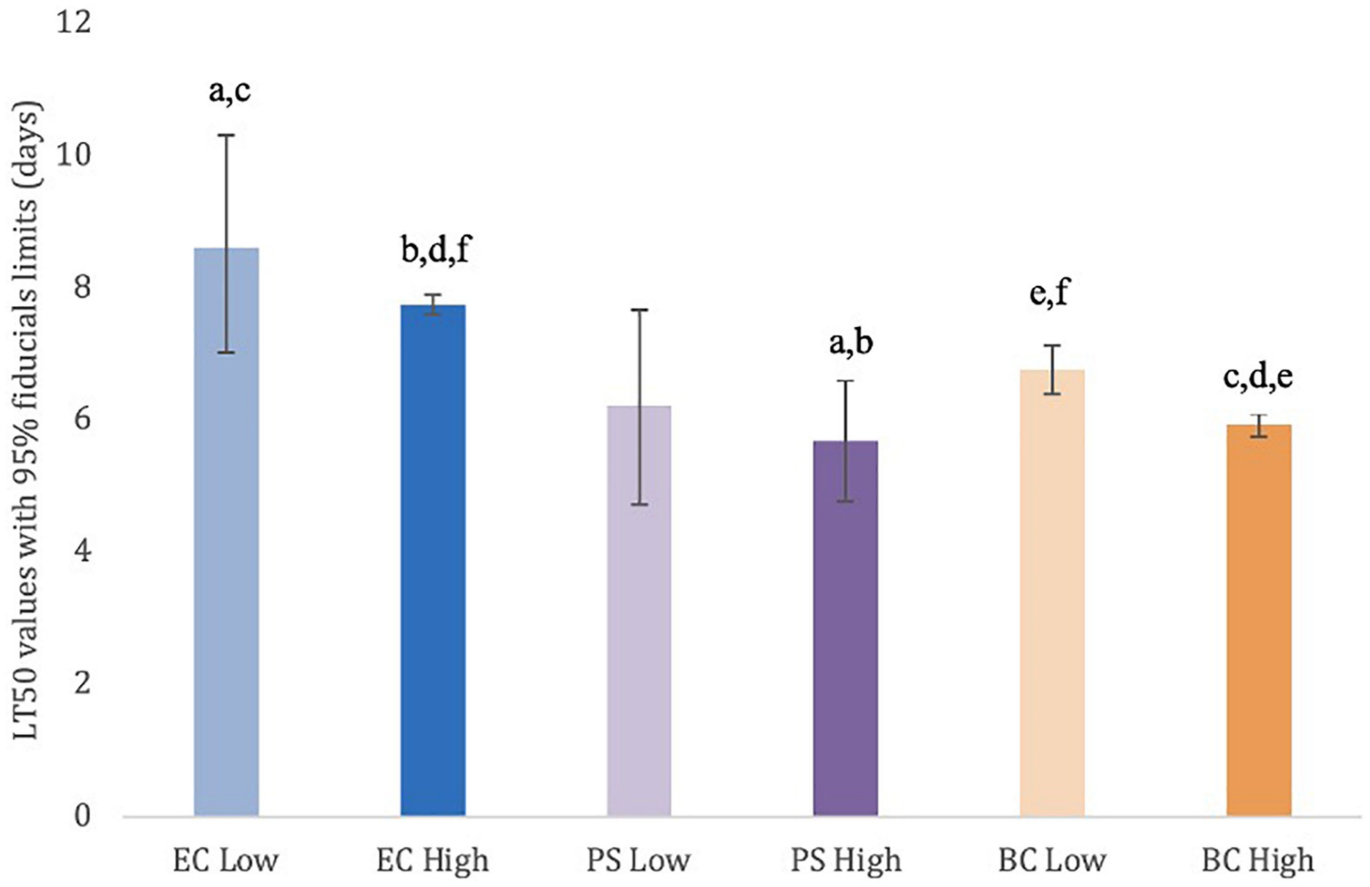
Lethal time 50 (days) with 95% fiducial limits for adult *D. reticulatum* when exposed to low and high rate treatments of *P. hermaphrodita.* Nematodes were paired with one of three designated bacterial food sources: *E. coli* OP50 (EC), *Pseudomonas* sp. (PS), and the original bacterial community (BC) associated with the nematodes. Treatment data were corrected for control mortality using Abbott’s formula. Bars with the same lowercase letter indicate a statistically significant difference (*p* < 0.05) between treatments. These difference exists when there is no overlap between the fiducial limits. Lethal time 50 values (95% fiducial limits): EC low rate = 8.61 (7.01–10.31), EC high rate = 7.74 (7.60–7.88), PS low rate = 6.23 (4.73–7.66), PS high rate = 5.68 (4.78–6.59), BC low rate = 6.77 (6.39–7.13), BC high rate = 5.92 (5.75–6.09).

Slugs that were exposed to the PS high dose treatment (LT50: 5.68 days) survived for significantly (*p* < 0.05) fewer days than those treated with both the EC high (LT50: 7.74 days) and low (LT50: 8.61 days) rates (Figure 1). For BC high (LT50: 5.92 days), slugs survived significantly (p < 0.05) fewer days than BC low (LT50: 6.77 days) and both EC rates. Lastly, slugs exposed to BC low survived significantly fewer days than those treated with EC high (Figure 1).

### Bacterial 16S rRNA sequence data

After quality filtering and removing chimeric sequences, we obtained a total of 10,485,882 reads, with an average of 582,549 reads and a median depth of 349,960 reads per sample. 2,938 ASVs (i.e., taxa) were identified. The decontamination procedure removed 131 ASVs, leaving 2,807 unique ASVs for subsequent analyses. The components of the removed contaminants are shown in Supplementary Figure S1. The filtered data set then contained 10,422,195 reads in total, with an average depth of 579,010 reads and a median 343,942 reads per sample. Details of the number of quality-controlled reads obtained in each sample, as well as the total number of reads, median, and mean values of reads per nematode-bacterial combination were listed in Supplementary Table S2.

### Microbiome shifts before and after slug infection experiment

**Figure 2:**
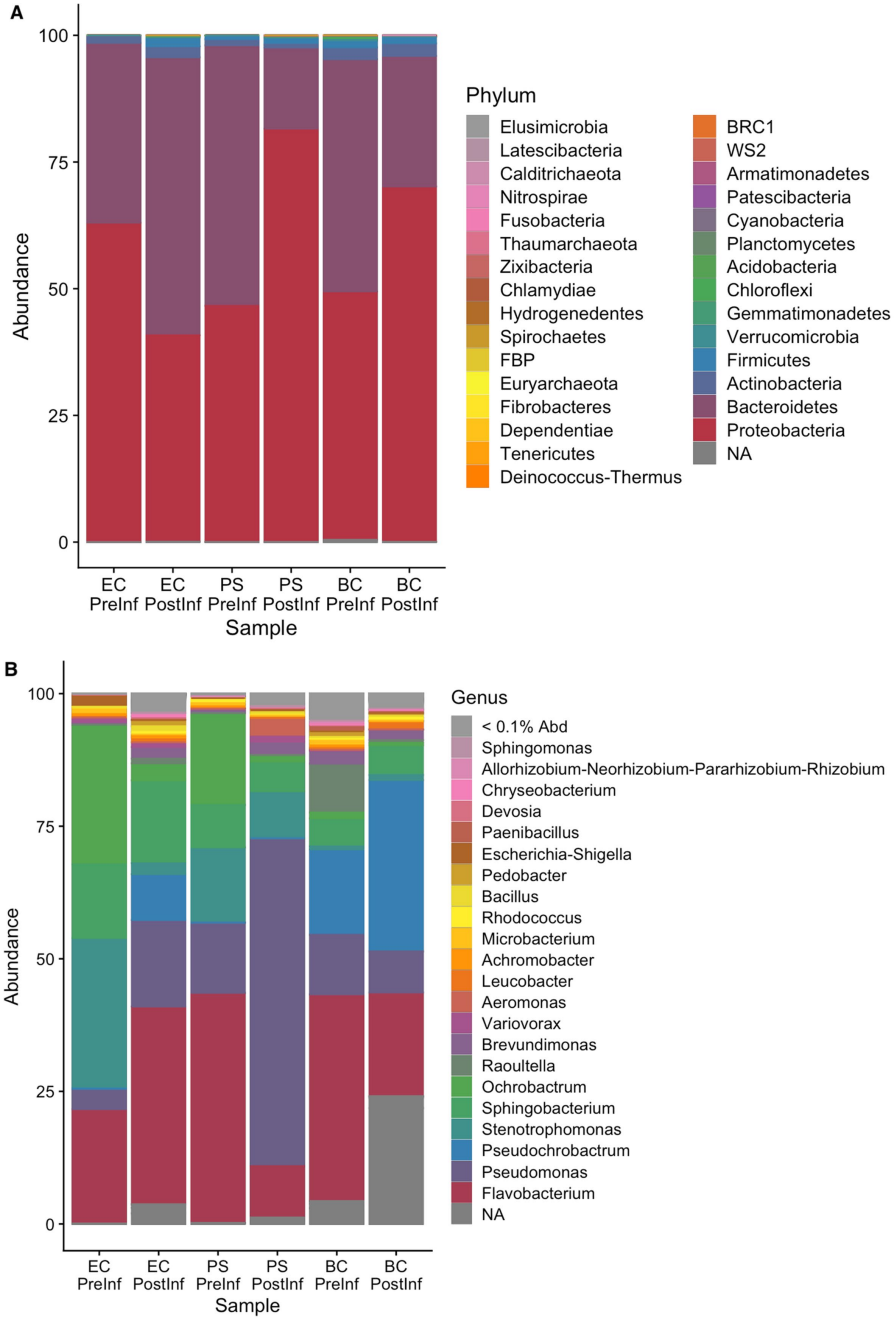
Composition of bacterial communities associated with *P. hermaphrodita* in each sample group on the **(A)** phylum and **(B)** genus level.

The number of different genera detected in each sample included in the analysis is listed in Table 1. On the Phylum level, the majority of bacterial components in all samples were identified as members of the phyla Bacteroidetes and Proteobacteria. We observed a shift in composition towards Proteobacteria in the BC-PostInf and PS-PostInf samples compared to their PreInf counterparts, while in the EC-PostInf samples, there was a shift towards Bacteroidetes (Figure 2A). In general, the number of genera in all samples increased almost fourfold after infection. The abundance of the main genera also altered remarkably between PreInf and PostInf samples (Figure 2B).

**Table 1:** The number of unique genera detected in each sample.

Bacterial treatment	Sample (before infection)	No. of genera	Sample (after infection)	No. of genera
*E. coli*	EC-PreInf-1	30	EC-PostInf-1	108
	EC-PreInf-2	31	EC-PostInf-2	185
	EC-PreInf-3	32	EC-PostInf-3	80
*Pseudomonas* sp.	PS-PreInf-1	22	PS-PostInf-1	108
	PS-PreInf-2	27	PS-PostInf-2	115
	PS-PreInf-3	24	PS-PostInf-3	107
Bacterial complex	BC-PreInf-1	66	BC-PostInf-1	130
	BC-PreInf-2	36	BC-PostInf-2	182
	BC-PreInf-3	35	BC-PostInf-3	144

In *P. hermaphrodita*’s original microbiome (i.e., the BC-PreInf community), 15 phyla were identified with Bacteroidetes being the most abundant phylum, followed by Proteobacteria. On the Genus level, the most abundant groups were *Pseudochrobactrum*, *Flavobacterium*, *Raoultella*, and *Pseudomonas* out of the 82 genera observed (Figure 2B). Details of relative taxa abundance on the Phylum and Genus level in the BC-PreInf communities were shown in Supplementary Table S3A.

**Figure 3:**
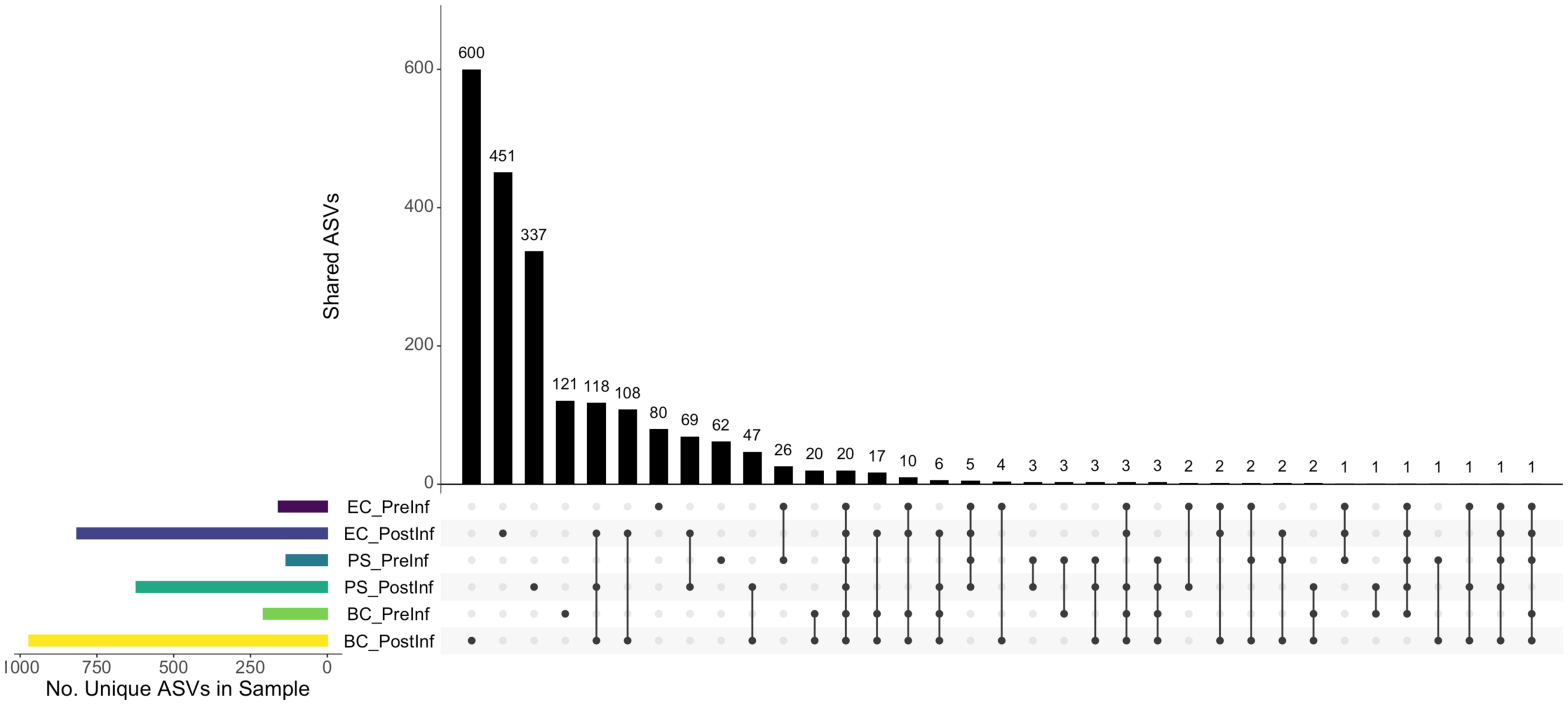
The ASV components present in each of the sample sets. Horizontal bars (left) indicate the number of unique ASVs found in each sample group; black dots (bottom) demonstrate the presence of ASVs in one or multiple samples; and the corresponding vertical bars indicate the number of ASVs with the presence described by the dots.

The numbers of ASVs unique to specific sample sets and shared among sample sets were illustrated in Figure 3. In general, the number of ASVs unique to a single sample set was higher than those shared between different sets. The BC-PostInf samples had the largest number of unique ASVs (i.e., 600) that were not shared with any other type of samples, followed by the EC-PostInf and PS-PostInf (451 and 337 ASVs, respectively). Comparing PreInf and PostInf samples, the BC-PreInf and BC-PostInf communities shared 82 common ASVs, while PS-PreInf and PS-PostInf shared 36 ASVs, and 44 ASVs were shared between EC-PreInf and EC-PostInf samples.

### Patterns of change in *α*-diversity

**Figure 4:**
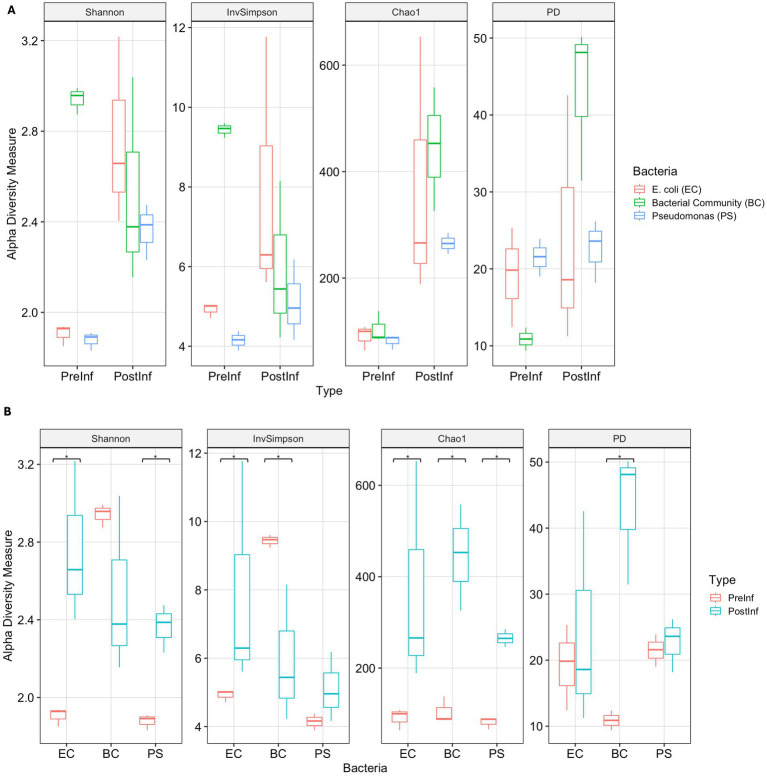
*α*-diversity indices Shannon, Inverse Simpson, Chao1 and Faith’s Diversity of samples grouped by **(A)** Time of collection and **(B)** Bacterial treatment. Significant differences of the metrics between pre- and post-infection of each bacterial treatment are indicated with the significant code: (****) *p* < = 0.0001, (***) *p* < = 0.001, (**) *p* < = 0.01, (*) *p* < = 0.05.

We hypothesized that slug infection would alter the diversity of the bacterial community within *P. hermaphrodita* nematodes, and that these changes would be influenced by the pre-infection bacterial associate. To test this, we evaluated *α*-diversity of the nematodes’ bacterial communities using four metrics: an estimator of species richness (Chao1), two metrics of species richness and evenness (Shannon index, Inverse Simpson), and a phylogeny-based measure (Faith’s Phylogenetic Diversity - PD) (Figure 4).

Bacterial community diversity significantly increased following nematode infection when comparing PreInf and PostInf samples across all three bacterial combinations. Specifically, the Chao1 index showed a significantly higher median across all PostInf samples compared to PreInf (Mann–Whitney U test, *p* < 0.001) (Figure 4A), indicating a clear increase in the number of ASVs and higher total species richness within the PostInf samples. Faith’s PD also showed trends toward higher diversity in terms of species richness and phylogenetic diversity post-infection, though this difference was only suggestive (Mann–Whitney U, *p* = 0.06).

Examining each treatment individually, bacteria-nematode combinations exhibited distinct diversity patterns following infection (Figure 4B). The EC microbiomes showed significant increases in Chao1 richness, Inverse Simpson index, and Shannon diversity following slug infection (Wilcoxon test, *p* = 0.05). These changes were consistent with each other and indicated a higher total number of bacterial taxa (species richness) and higher evenness among taxa members post-infection, though insignificant Faith’s PD result suggested that there was no evidence of a shift in phylogenetic diversity of taxa compositions. In BC PostInf samples, Faith’s PD and Chao1 richness increased significantly (Wilcoxon test, *p* = 0.05), while the Inverse Simpson index decreased (Wilcoxon test, *p* = 0.05), demonstrating an increase in phylogenetically diverse taxa alongside reduced evenness, which possibly resulted from the emergence of many new low-abundance taxa compared to pre-infection samples. For the *Pseudomonas-*enriched communities, the PS-PostInf samples demonstrated strong evidence of increases in Shannon diversity and Chao1 index (Wilcoxon test, *p* = 0.05) compared to PS-PreInf, indicating that the bacterial communities in the *Pseudomonas*-enriched treatment developed greater species richness and community diversity following slug infection.

As expected, *α*-diversity differed significantly among microbiomes from the three bacteria-nematode combinations prior to infection (Kruskal-Wallis test, *p* = 0.05), with EC-PreInf and PS-PreInf communities showing lower diversity than BC-PreInf due to their more defined, single taxon-enriched starting compositions. However, after the infection assay, *α*-diversity indices between the three bacteria-nematode combinations did not differ significantly (Kruskal-Wallis test, *p* = 0.30). This suggests that the infection process introduced additional taxa to the bacterial communities, potentially reaching a similar *α*-diversity equilibrium regardless of the initial bacterial associate.

### Patterns of change in *β*-diversity

Given the distinct starting bacterial associates, we hypothesized that bacterial community structures would differ significantly among the three bacteria-nematode combinations (EC, PS, and BC). We also predicted that going through the slug infection process would alter the bacterial community composition within each treatment, leading to detectable shifts in community structure between pre- and post-infection samples. To evaluate the dissimilarity in bacterial community composition among samples from the three combinations before and after the infection assay, we applied four complementary β-diversity indices: an abundance-weighted metric (Bray–Curtis dissimilarity), a presence-absence index (Jaccard distance), and two phylogenetically-based indices with and without weighting on taxa abundances (weighted and unweighted UniFrac, respectively).

**Figure 5:**
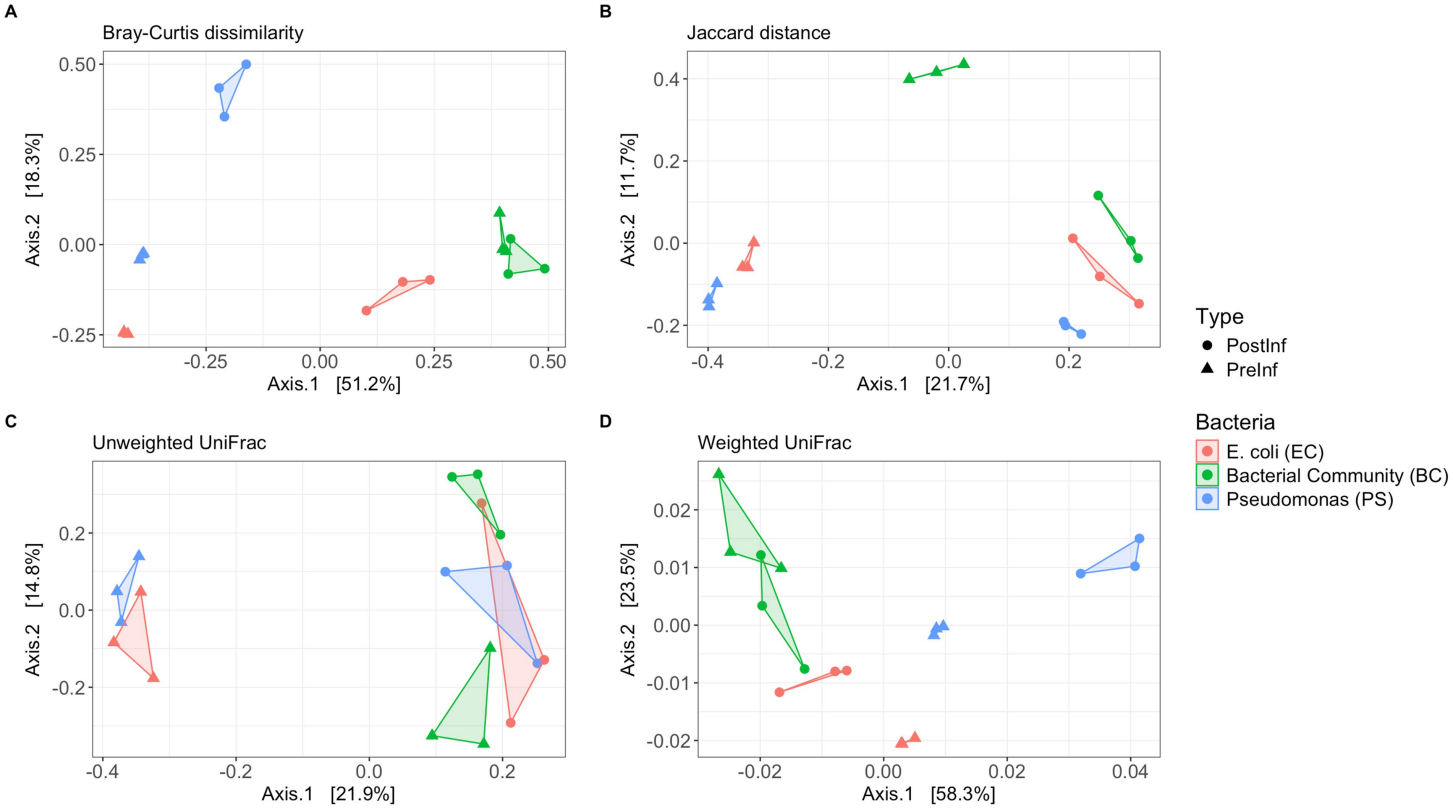
*β*-diversity indices of the microbiome samples. Principal coordinate analysis (PCoA) with **(A)** Bray–Curtis dissimilarity, **(B)** Jaccard distance, **(C)** Unweighted UniFrac distance, and **(D)** Weighted UniFrac distance. Samples are colored by the type of bacterial treatment and shaped by the time of collection.

Bray-Curtis analysis results showed a clear pattern of distinct sample clustering (Figure 5). The bacterial community structures were significantly different among the EC, PS, and BC sample sets (PERMANOVA, *p* = 0.001, R2 = 0.541). This result was robust to Jaccard distance (PERMANOVA, p = 0.001) and weighted UniFrac indices (PERMANOVA, p = 0.001). However, unweighted UniFrac showed only marginal significance (PERMANOVA, *p* = 0.06). These results suggest that microbiomes from distinct bacterial conditions significantly differed in species composition, phylogenetic distribution, and community structure. Since unweighted UniFrac considers only the presence or absence of taxa and is particularly sensitive to differences in rare lineages, while weighted UniFrac incorporates taxa abundance information, the significant differences shown by weighted UniFrac but not unweighted UniFrac suggest that the differences among the three bacterial treatments were mainly driven by variations in the relative abundances of common taxa, rather than differences in the presence or absence of rare, phylogenetically unique species.

Comparing pre-infection and post-infection samples, Bray–Curtis dissimilarity, Jaccard distance, and unweighted UniFrac indicated a significant compositional difference (PERMANOVA, *p* = 0.02, *p* = 0.01, *p* = 0.001, respectively); however, weighted UniFrac did not detect significant changes (*p* = 0.25). The shift detected by unweighted UniFrac but not by abundance-weighted UniFrac suggests that the differences in community composition and structure pre- and post- infection mainly resulted from changes in rarer taxa, which contribute less to the abundance-weighted phylogenetic distance calculations.

### Pseudomonas ASV expansion after slug infection in the PS samples

**Figure 6:**
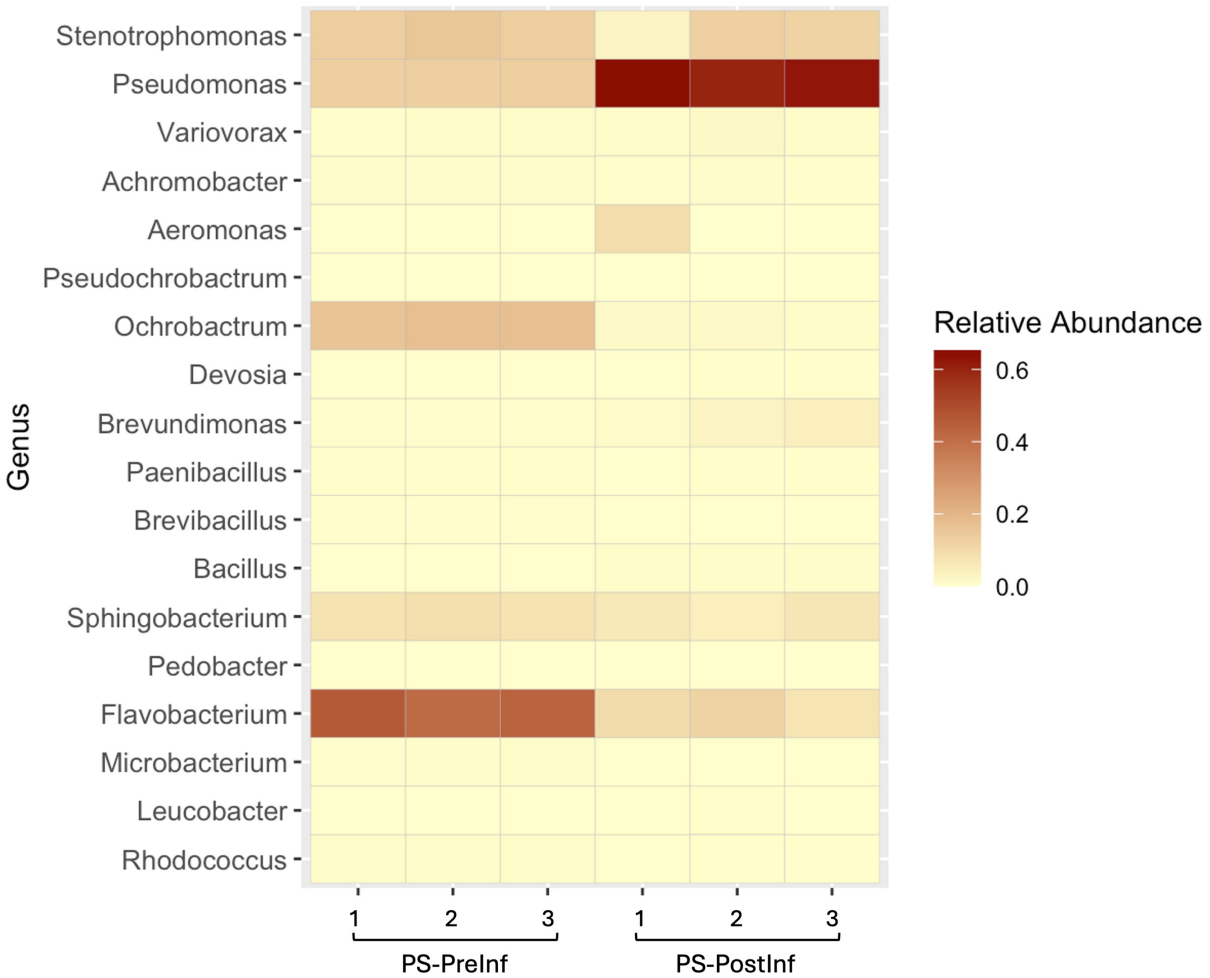
Heatmap of relative abundances for the top 20 most abundant bacterial genera across all biological replicates of the *Pseudomonas*-enriched treatment. Color intensity represents relative abundance values, with beige indicating lower and dark red showing higher abundances.

We analyzed the ASV components of the PS microbiome, with specific focus on those deriving from *Pseudomonas* spp., to evaluate the potential effects of the infection process on this group of bacteria (Figure 6). The microbiome of PS-PostInf samples only shared a total of 36 ASVs with PS-PreInf, four of which were identified as belonging to the genus *Pseudomonas*. The relative abundance of these four ASVs in PS-PreInf samples was 13.23% of the total, and expanded 4.5-fold to 59.58% in PS-PostInf samples. Among these four ASVs, one was present in all three bacterial-nematode combinations sampled both before and after infection, and displayed an increase of 7.5, 3.6, and 3.2% in EC, PS, and BC communities, respectively. The three other *Pseudomonas* ASVs were detected only in the microbiomes of PS and BC nematodes, both before and after the infection assay; they were absent from all EC samples. While their abundances all increased after the course of infection in PS samples - by 6.9, 30.5, and 5.5% - there was a consistent decrease of these ASVs by 1.38, 4.13, and 0.73% from BC-PreInf to BC-PostInf samples. Two *Pseudomonas* ASVs present exclusively in PS-PreInf accounted for just 0.004% abundance; fourteen ASVs unique to the PS-PostInf samples had a total relative abundance of 1.11%.

## Discussion

We performed a 16S rRNA-based analysis of the *P. hermaphrodita* microbiota sampled immediately before and after infection of grey field slug hosts. We hypothesized that varying microbiomes associated with *P. hermaphrodita* would differentially impact the nematode’s ability to infect slugs, either through direct effects (e.g., direct involvement of bacteria in slug mortality) or indirect mechanisms (e.g., effects on nematode nutritional metabolism or similar). We observed that slug survival varied significantly depending on the bacterial partners associated with the nematodes prior to infection: Slugs exposed to nematodes reared on the neutral *E. coli* survived significantly longer than those exposed to high dosed nematodes reared on either *Pseudomonas* or the original bacterial community. Once slug mortality began, on average, nematodes reared on *Pseudomonas* achieved 100% mortality within only 3 days, faster than BC nematodes (5 days) and EC nematodes (10 days). These findings support our hypothesis and suggest that the starting bacterial associate influences the nematode’s pathogenicity and killing efficacy against slugs.

### Bacterial communities associated with *P. hermaphrodita*

We also hypothesized that nematode-associated microbiomes would change significantly from pre-to post-infection samples. Host infection might act as a selection pressure on the bacterial community, favoring the bacteria that can better contribute to the nematode’s virulence, spread, reproduction, and killing efficacy. Furthermore, recent research has shown differential microbiome responses between nematode-susceptible and nematode-resistant host gastropod species (Sheehy et al., 2022).

The microbiome samples of all the EC, PS, and BC samples showed complex taxa (ASVs) compositions. The species richness and diversity of EC-PreInf and PS-PreInf communities were higher than our expectations, given that the nematodes had been bleached with hypochlorite, washed thoroughly with water and salt solutions, and reared on pure cultures of *E. coli* or *Pseudomonas* sp. before the infection assay. The intended bacteria were present in the samples collected before infection trials; however, *Pseudomonas* sp. and *E. coli* accounted for a relatively minor component of the community by the time the infection experiments began. Several factors likely contributed to the unexpectedly high taxonomic diversity observed in the EC and PS samples, including environmental bacteria in the lab, certain bacteria surviving the bleaching procedures, and the possibility that there are unknown bacterial endosymbionts associated with *P. hermaphrodita* as has been reported in other nematode systems (Brown et al., 2018; Mobasseri et al., 2019).

Before the infection trial, fifteen different phyla and 82 genera were observed in the BC-PreInf samples, with *Pseudochrobactrum, Flavobacterium, Raoultella*, and *Pseudomonas* being the four most abundant taxa in the community. These four genera have all been reported in association or rivalry with nematodes, for example, *Pseudochrobactrum* spp. with the EPN *Steinernema* (Ogier et al., 2020), *Raoultella* spp. with the root-knot and pine wood nematodes (Liu et al., 2019), *Flavobacterium* spp. with the insect-parasitic nematode *Rhabditis blumi* (Park et al., 2011), the soybean cyst nematode *Heterodera glycines* (Nour et al., 2003), the oriental beetle pathogenic nematode *Butlerius* spp. (Yi et al., 2007), and *Pseudomonas* spp. with *C. elegans* (Pees et al., 2024). The high-abundance components in BC-PreInf samples are relatively similar to those of *C. elegans*, whose dominant genera are *Pseudomonas, Stenotrophomonas, Ochrobactrum, Sphingomonas*, and unclassified Enterobacteriaceae (*Raoultella* is a representative of this family) (Dirksen et al., 2016; Zhang et al., 2017). *Stenotrophomonas, Ochrobactrum*, and *Sphingomonas* spp. were all detected in the top 10 most abundant genera in our BC-PreInf bacterial community.

The complex nature of the bacterial community structure and the results of slug mortality during the infection assay indicated that *P. hermaphrodita* could carry out slug infection with various bacterial partners regardless of the primary bacterial food source, although at different rates. This observation agrees with a previous finding that the nematode is able to associate with non-specific and complex microbial assemblages (Rae et al., 2010). However, slug-killing efficacy clearly varied between different bacteria-nematode combinations. BC-cultured nematodes were the first out of the three treatments to cause significant slug mortality on Day 5, followed by the PS nematodes on Day 6, and lastly, the EC nematodes, starting Day 10. However, the nematodes treated with *Pseudomonas* took only 3 days to kill all slugs and were the first to cause 100% slug mortality, followed by BC nematodes (5 days) and EC nematodes (10 days). This accelerated killing rate suggests that *Pseudomonas* spp. may influence the nematode’s pathogenicity, possibly by assisting *P. hermaphrodita*’s virulence, acting as an opportunistic pathogen, and/or providing a favorable food source that benefits the nematode’s growth and development, thereby enhancing infection and killing efficacy.

Analysis of ASVs in both the PS-PreInf and PS-PostInf sample sets from the *Pseudomonas* treatment revealed four common ASVs belonging to the genus *Pseudomonas* (see Results). In the PS treatment, the relative abundance of these four *Pseudomonas* ASVs expanded 4.5-fold from PS-Pre-Inf to PS-PostInf samples, accounting for approximately 60% of total abundance of all taxa in the community. Among these four ASVs, we detected the presence of one particular ASV in all sample sets, whose relative abundance consistently increased after the course of infection in all three bacterial treatments. This suggests that this specific ASV might be stably retained by the nematode and could be important to the slug infection activity. The abundance of the three other *Pseudomonas* ASVs also expanded after infection in PS-PostInf samples. However, in BC-PostInf samples, *Pseudomonas* ASVs did not exhibit the same expansion pattern. The lack of *Pseudomonas* expansion in BC-PostInf samples, despite the apparent pathogenic effects, suggests that the potential role of *Pseudomonas* spp. in pathogenicity may be dependent upon the microbiome context. In the PS treatments, which have lower species complexity than BC, the expansion of these ASVs may suggest a role in pathogenicity. This pattern is not observed in BC, potentially due to interactions or competition with other bacterial taxa that interfere with *Pseudomonas* function in the infection process, preventing it from achieving the dominance necessary to enhance nematode pathogenicity as observed in the *Pseudomonas*-enriched cultures.

### Bacterial community shifts after slug infection

In general, we found that *α*-diversity indices were higher in all post-infection samples compared to pre-infection samples, especially in the *Pseudomonas* and bacterial community treatments. For BC-PostInf samples, Chao1 and Faith’s PD *α*-diversity indices increased significantly, which may indicate the appearance of low-abundance taxa during the course of infection, and also suggest that the diversity in the BC PostInf microbiome results not just from the presence of a few highly diverse taxonomic groups, but rather from a broader range of phylogenetically different taxa. Indeed, for example, the total number of different genera in the BC microbiomes more than tripled after infection, however, the composition of the top 20 most abundant taxa remained stable. Likewise, for the *Pseudomonas-*enriched treatment, we reported significant increases in Chao1 and Shannon indices after the assay, indicating a spike in rare taxa post-infection, although composition analysis pointed to the dominance of *Pseudomonas* taxa in the community.

### Microbial community differs by type of bacterial treatment and time of collection

We found that the difference between samples from different bacteria-nematode combinations was significant for Bray–Curtis dissimilarity, Jaccard distance, and weighted UniFrac metrics, but not for unweighted UniFrac. This reveals that between bacterial combinations, the communities comprehensively differed in structure, taxa composition, and phylogenetic distribution. The non-significant unweighted UniFrac result suggests that the same major phylogenetic groups were present across all bacterial treatments. However, the significant weighted UniFrac result implies that the relative abundance of these shared phylogenetic lineages was drastically different. In other words, the microbiomes in different bacterial combinations were not reshaped by the loss or gain of entire phylogenetic branches, but rather by profoundly altering which taxa were dominant.

Meanwhile, samples collected before and after infection show a significant difference according to the unweighted UniFrac but not the weighted UniFrac metric. This pattern indicates that the shift in community composition pre- and post-infection mainly resulted from the gain or loss of low-abundance taxa, while the most abundant taxa remained relatively stable. This observation is in concordance with the findings of the *α*-diversity analyses discussed above.

## Conclusion

In this study, we assessed the pathogenicity of *P. hermaphrodita* in association with different bacteria and documented compositional changes in the nematode’s microbiome before and after slug infection. We observed substantial increases in species richness across treatments; however, the composition of the most abundant taxa pre-infection remained largely stable from pre- to post-infection. We also detected four specific ASVs of the genus *Pseudomonas*, whose relative abundance expanded remarkably after infection in the *Pseudomonas-*enriched communities, but not in the nematode’s natural bacterial communities. These finding suggests competitive or certain synergistic effects within the original bacterial community that warrant further investigation to fully understand the complex relationships between nematode infection, bacterial community composition, and pathogenicity outcomes.

## Data Availability

The data product files presented in this study are deposited in a Zenodo archive, publicly available here: https://doi.org/10.5281/zenodo.15658705.
