# Supplementary material for: Microbiome dynamics associated with the infection of grey field slugs by the biocontrol nematode Phasmarhabditis hermaphrodita
Source: Front Microbiol. 2025 Aug 20;16:1619231. doi: 10.3389/fmicb.2025.1619231 (PMC12405279; doi:10.3389/fmicb.2025.1619231)
Supplement: Supplementary file 1 [file Data_Sheet_1.pdf]

Supplementary material

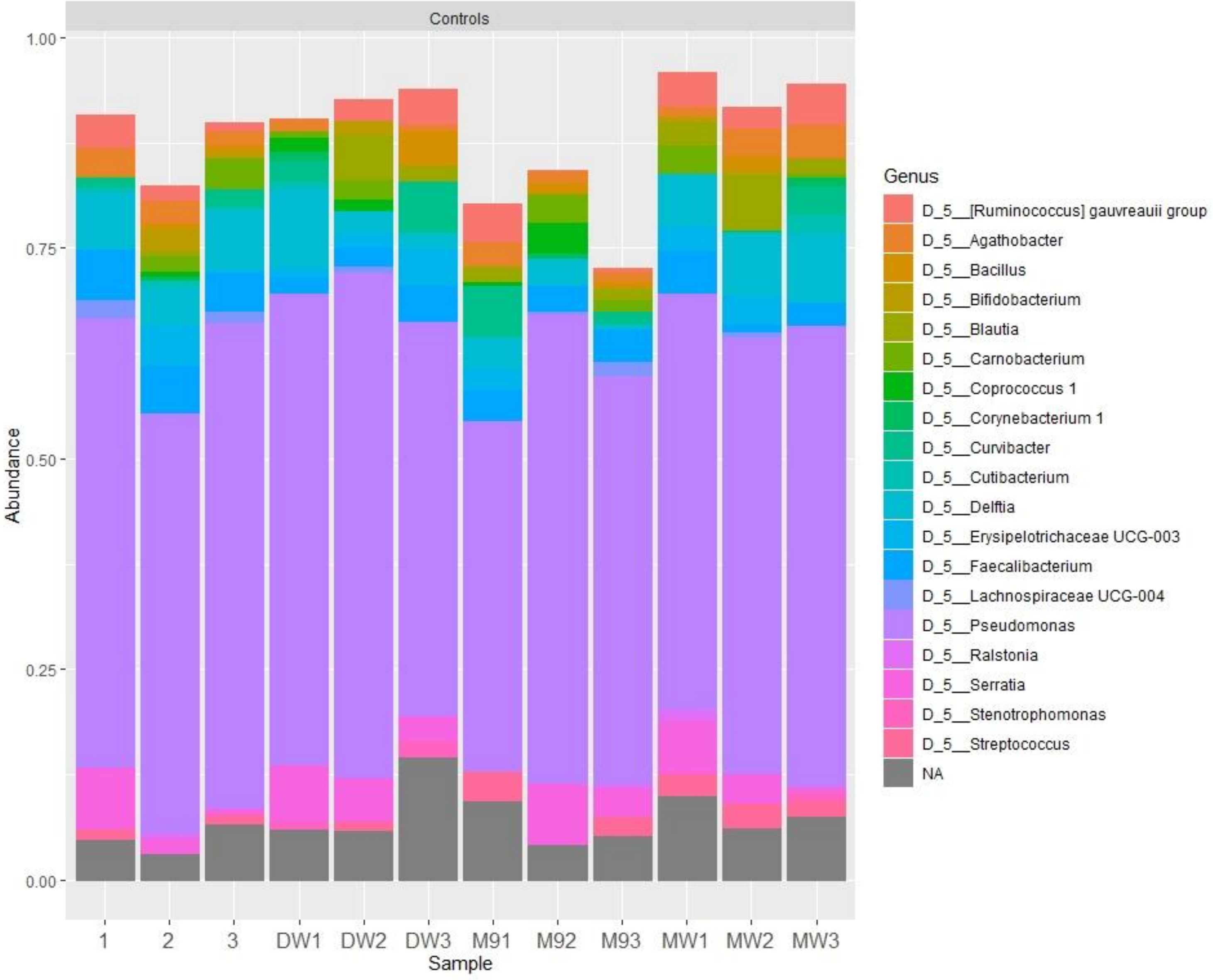

Figure S1. Composition of putative ASVs contaminants removed from ASV table. ASVs were considered a contaminant if they were more prevalent in negative controls than in positive samples and/or their frequency significantly varied inversely with sample DNA concentration (probability threshold $p < 0.1$). Negative control sample abbreviations: 1-3: DNA Extraction kit blank, DW1-DW3: distilled water used during nematode washing step, MW1-MW3: distilled water used during infection assay step, M91-M93: M9 buffer used in nematode washing.

Table S1. Naming scheme of samples

**Bacterial treatment**	**Time of collection**	**Sample name**
E. coli-enriched samples	before infection	EC-PreInf
	after infection	EC-PostInf
Pseudomonas sp. -enriched samples	before infection	PS-PreInf
	after infection	PS-PostInf
Complex bacterial community	before infection	BC-PreInf
	after infection	BC-PostInf

Table S2. Quality-controlled read counts in decontaminated samples.

Sample group	Replicate	No. of reads	Total reads in each combination	Median value in each combination	Mean value in each combination
EC-PreInf	1	604,813	4,087,108	490,705	681,185
	2	1,610,879			
	3	1,091,611			
EC-PostInf	1	224,670			
	2	376,596			
	3	178,539			
BC-PreInf	1	153,427	2,572,446	231,484	428,741
	2	153,680			
	3	100,672			
BC-PostInf	1	309,288			
	2	1,005,733			
	3	849,646			
PS-PreInf	1	641,501	3,762,641	453,176	627,107
	2	1,198,645			
	3	1,231,796			
PS-PostInf	1	264,851			
	2	250,356			
	3	175,492			

Table S3: **(A)** Phylum composition and relative abundance of the bacterial community associated with *P. hermaphrodita* before infection (samples BC-PreInf). Phyla were sorted from the highest to lowest mean abundance in samples.

Phylum	meanRA	sdRA	minRA	maxRA
D_1__Bacteroidetes	0.022711	0.05372	1.30E-05	0.216386
D_1__Proteobacteria	0.0099	0.025887	1.99E-05	0.195276
D_1__Gemmatimonadetes	0.002883	0.004853	7.81E-05	0.010142
D_1__Firmicutes	0.002073	0.003461	1.95E-05	0.012119
D_1__Chloroflexi	0.001788	NA	0.001788	0.001788
D_1__Planctomycetes	0.001664	0.001337	3.90E-05	0.003268
D_1__Acidobacteria	0.001592	0.000449	0.001222	0.002245
NA	0.001585	0.002498	1.30E-05	0.006981
D_1__Actinobacteria	0.001375	0.001653	3.26E-05	0.006019
D_1__Verrucomicrobia	0.001323	0.001488	0.000169	0.003447
D_1__WS2	0.000864	NA	0.000864	0.000864
D_1__Armatimonadetes	0.000808	NA	0.000808	0.000808
D_1__Patescibacteria	0.000505	2.30E-05	0.000489	0.000521
D_1__Cyanobacteria	0.000192	0.000169	5.96E-05	0.000437
D_1__Deinococcus-Thermus	4.97E-05	NA	4.97E-05	4.97E-05

(B) Genus composition of the natural bacterial community associated with *P. hermaphrodita*. Genera were sorted from the highest to lowest mean abundance in samples.

Genus	meanRA	sdRA	minRA	maxRA
D_5__Pseudochrobactrum	0.069449	0.085976	0.002215	0.195276
D_5__Flavobacterium	0.045221	0.077108	1.30E-05	0.216386
D_5__Raoultella	0.01917	0.020054	0.000404	0.062018
D_5__Pseudomonas	0.018958	0.027937	4.55E-05	0.110072
D_5__Brevundimonas	0.010851	0.011081	0.000467	0.029283
D_5__Sphingobacterium	0.007877	0.00789	0.000235	0.026679
D_5__Pedobacter	0.005992	0.002071	0.003795	0.009097
D_5__wb1-P19	0.005553	NA	0.005553	0.005553
D_5__Ochrobactrum	0.005453	0.003768	0.001662	0.011023
D_5__Bauldia	0.005066	NA	0.005066	0.005066
D_5__Paenibacillus	0.004858	0.004821	0.000469	0.012119
D_5__Achromobacter	0.004599	0.002172	0.001597	0.00691
D_5__Kaistia	0.004247	0.004221	0.00058	0.00886
NA	0.003932	0.007775	1.30E-05	0.033866
D_5__Sphingomonas	0.003857	0.003819	0.000111	0.009262
D_5__ADurb.Bin063-1	0.003447	NA	0.003447	0.003447
D_5__Microbacterium	0.003383	0.002822	0.000372	0.006019
D_5__Rhodococcus	0.003253	0.002015	0.000111	0.00509
D_5__Stenotrophomonas	0.003141	0.004696	1.99E-05	0.012148
D_5__Allorhizobium-Neorhizobium-Pararhizobium-Rhizobium	0.003072	0.001925	0.000891	0.005275

D_5__Rhodoferax	0.002959	NA	0.002959	0.002959
D_5__uncultured	0.002167	0.002845	3.90E-05	0.010142
D_5__Comamonas	0.002157	0.001104	0.001356	0.003416
D_5__Anaerococcus	0.002007	NA	0.002007	0.002007
D_5__Bacteroides	0.001973	0.002716	5.21E-05	0.003894
D_5__Marmoricola	0.001828	0.001888	0.000104	0.00449
D_5__uncultured soil bacterium	0.001788	NA	0.001788	0.001788
D_5__Herminiimonas	0.001429	0.000767	0.000886	0.001972
D_5__Chryseobacterium	0.001421	NA	0.001421	0.001421
D_5__Caulobacter	0.001351	0.001301	5.96E-05	0.003084
D_5__Propionivibrio	0.001297	NA	0.001297	0.001297
Ambiguous_taxa	0.001281	0.000945	0.000613	0.001949
D_5__Oikopleura dioica	0.001245	NA	0.001245	0.001245
D_5__RB41	0.001222	NA	0.001222	0.001222
D_5__Porphyrobacter	0.001162	NA	0.001162	0.001162
D_5__uncultured bacterium	0.001155	0.001066	0.000489	0.003258
D_5__Terrimonas	0.001108	NA	0.001108	0.001108
D_5__Nakamurella	0.001108	NA	0.001108	0.001108
D_5__Nesterenkonia	0.001088	NA	0.001088	0.001088
D_5__Rathayibacter	0.001036	NA	0.001036	0.001036
D_5__Crossiella	0.000939	NA	0.000939	0.000939
D_5__Leucobacter	0.000917	0.000763	0.00013	0.002781
D_5__Lachnoclostridium	0.000867	NA	0.000867	0.000867
D_5__Pir4 lineage	0.00072	0.000963	3.90E-05	0.001401

D_5__Variovorax	0.000719	0.000572	0.000137	0.001623
D_5__Friedmanniella	0.000704	NA	0.000704	0.000704
D_5__Nordella	0.000691	NA	0.000691	0.000691
D_5__Devosia	0.000677	0.000596	0.000104	0.001744
D_5__Brevibacillus	0.00062	0.000738	9.78E-05	0.001464
D_5__Phreatobacter	0.000602	0.000382	0.000332	0.000872
D_5__Shinella	0.000591	0.000626	0.000119	0.001301
D_5__Faecalibacterium	0.00055	0.000578	0.00015	0.001212
D_5__Nocardioides	0.000521	0.000516	0.000156	0.000886
D_5__Hydrogenophaga	0.000504	0.000621	6.52E-05	0.000944
D_5__Medicago truncatula	0.000437	NA	0.000437	0.000437
D_5__Arenimonas	0.00043	0.000378	0.000163	0.000697
D_5__Candidatus Udaeobacter	0.00043	NA	0.00043	0.00043
D_5__Geodermatophilus	0.000398	NA	0.000398	0.000398
D_5__Gemmatimonas	0.000365	NA	0.000365	0.000365
D_5__Streptococcus	0.000328	NA	0.000328	0.000328
D_5__Jatrophihabitans	0.000274	NA	0.000274	0.000274
D_5__Moheibacter	0.00026	NA	0.00026	0.00026
D_5__Roseburia	0.000254	0.000323	2.60E-05	0.000482
D_5__Sphingopyxis	0.000247	0.00023	8.47E-05	0.00041
D_5__uncultured Sphingomonadaceae bacterium	0.000235	NA	0.000235	0.000235
D_5__alphaI cluster	0.000235	NA	0.000235	0.000235
D_5__Coprococcus 1	0.000215	NA	0.000215	0.000215
D_5__Gaiella	0.000202	NA	0.000202	0.000202

D_5__Leptothrix	0.000182	NA	0.000182	0.000182
D_5__Prosthecobacter	0.000169	NA	0.000169	0.000169
D_5__Aeromicrobium	0.000163	NA	0.000163	0.000163
D_5__Rubellimicrobium	0.00015	NA	0.00015	0.00015
D_5__Candidimonas	0.000143	NA	0.000143	0.000143
D_5__Mesorhizobium	0.00014	0.000124	5.21E-05	0.000228
D_5__Delftia	7.81E-05	NA	7.81E-05	7.81E-05
D_5__metagenome	7.81E-05	NA	7.81E-05	7.81E-05
D_5__Rhodopseudomonas	6.52E-05	NA	6.52E-05	6.52E-05
D_5__Deinococcus	4.97E-05	NA	4.97E-05	4.97E-05
D_5__Mycobacterium	3.91E-05	NA	3.91E-05	3.91E-05
D_5__Conexibacter	3.26E-05	NA	3.26E-05	3.26E-05
D_5__Hyphomicrobium	2.60E-05	NA	2.60E-05	2.60E-05
D_5__Dorea	1.95E-05	NA	1.95E-05	1.95E-05
